# Supplementary material for: Hahahahaha, Duuuuude, Yeeessss!: A two-parameter characterization of stretchable words and the dynamics of mistypings and misspellings
Source: PLoS One. 2020 May 27;15(5):e0232938. doi: 10.1371/journal.pone.0232938 (PMC7252599; doi:10.1371/journal.pone.0232938)
Supplement: S1 Appendix — (PDF) [file pone.0232938.s001.pdf]

## Appendix A: Alternate Balance Measure

As a comparison to our normalized entropy measure for balance discussed in Sec. III B, we also compute an alternate normalized entropy measure, $H_{\mathrm{alt}}$, that measures balance from a different view.

To compute $H_{\mathrm{alt}}$, we first calculate the overall average stretch for each character as before, but now do so across all tokens at once. Then, we subtract one from each of these values and normalize them so they sum to 1 and can be thought of like probabilities. We then compute the normalized entropy, $H_{\mathrm{alt}}$, of these values as a measure of overall balance. $H_{\mathrm{alt}}$ is similar to $H$ in that if each character stretches the same on average, the normalized entropy is 1, and if only one character in the kernel stretches, the normalized entropy is 0. Again, higher entropy corresponds with more balanced words.

The difference is the view, and what is meant by 'on average'. For $H_{\mathrm{alt}}$, each token is weighted equally when calculating balance. Thus, this measure corresponds to the view of if one randomly samples tokens and looks at how balanced they are on average.

By contrast, for $H$, as calculated in Sec. III B, tokens are grouped by length, and then each group gets an equal weight regardless of the group size. This view looks at how well balance is sustained across lengths, and corresponds to sampling tokens by first randomly picking a length, and then randomly picking a token from all tokens of that length, and then looking at how balanced the sampled tokens are on average.

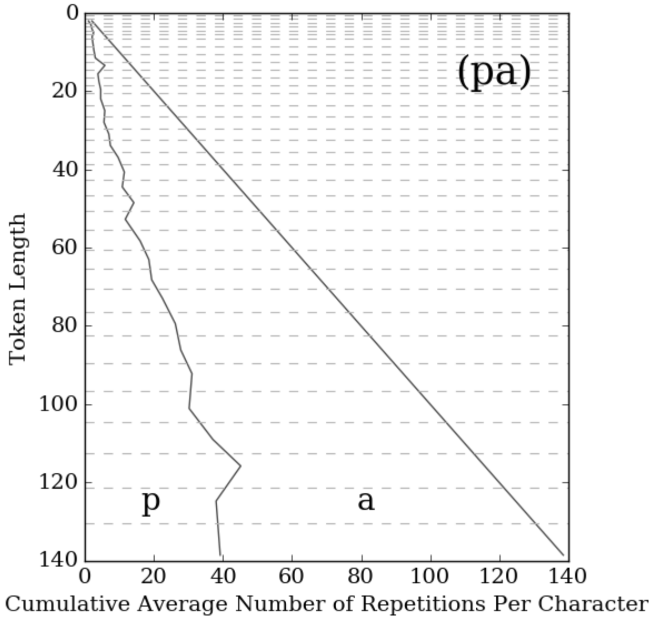


FIG. A1. Balance plot for the kernel (pa). See the Fig. 6 caption for plot details. Even though $H_{\mathrm{alt}} = 1.00000$ for (pa), this plot clearly shows perfect balance is not sustained as tokens increase in length.

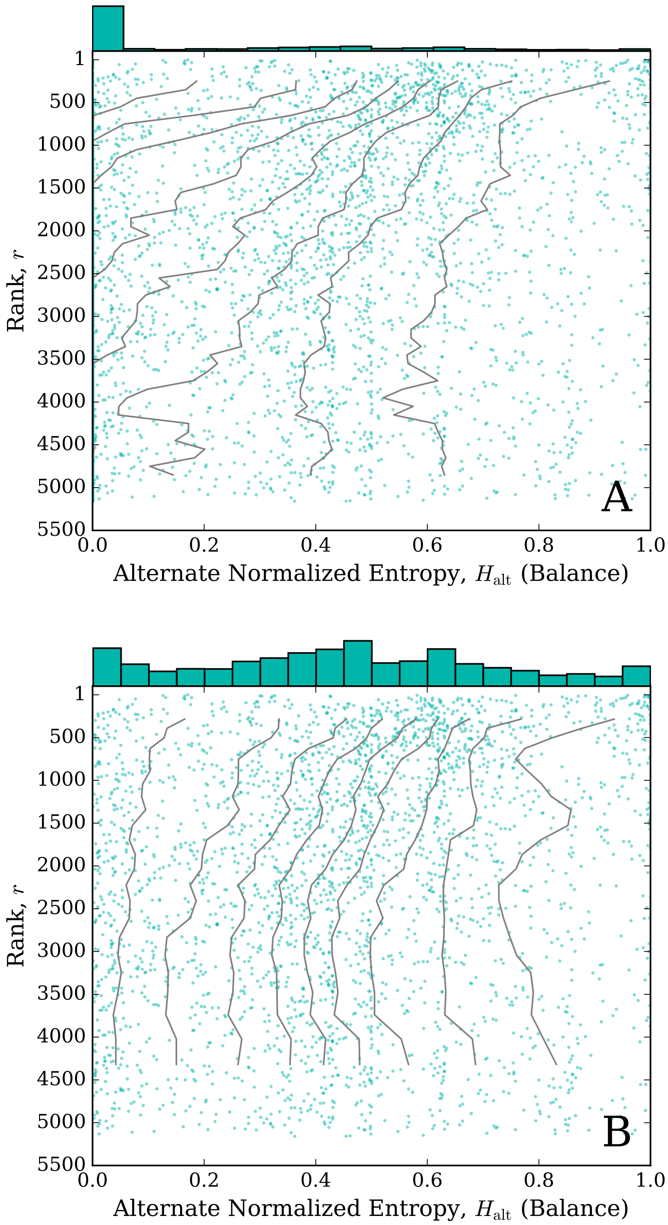


FIG. A2. Jellyfish plots for kernel balance based on an alternate entropy measure for (A) all kernels, and (B) excluding kernels with entropy exactly 0. Corresponding histograms are given at the top of each plot. Kernels are plotted vertically by their rank, $r$, and horizontally by their balance as given by an alternate normalized entropy, $H_{\mathrm{alt}}$, where larger entropy denotes increased balance. The deciles $0.1, 0.2, \ldots, 0.9$ are calculated for rolling bins of 500 kernels and are plotted as the 'tentacles'.

For example, for the kernel (pa), $H_{\mathrm{alt}} = 1.00000$, signifying nearly perfect balance. However, looking at the balance plot for (pa) in Fig. A1, we see that perfect balance is not sustained across lengths. Because most of the tokens are short, and short stretched versions of (pa) are

| | $H_{alt}$ | Kernel | Example token |
|---|---|---|---|
| 1 | 1.00000 | (ba) | baaaaaaaaaaa |
| 2 | 1.00000 | (pa) | ppppppppppppa |
| 3 | 1.00000 | (uo) | uouuuuuuuuuuuuu |
| 4 | 0.99998 | (pr) | prrrrrrrrrrr |
| 5 | 0.99998 | (du) | duduudduududududuuu |
| 6 | 0.99995 | (xa) | xaxaxaxaxxa |
| 7 | 0.99995 | (ai) | aaaaaaaaaaaaaaaai |
| 8 | 0.99993 | (he) | hehehheheheh |
| 9 | 0.99986 | (bi) | biiiiiiiii |
| 10 | 0.99985 | (wq) | wqwqwqwqwqw |

TABLE A1. Top 10 kernels by an alternate normalized entropy, $H_{alt}$.

| | $H_{alt}$ | Kernel | Example token |
|---|---|---|---|
| 1 | 0.00115 | [t][e][t]h | teeeeeeeeeth |
| 2 | 0.00119 | f[e]l[i]ng | feeeeeeling |
| 3 | 0.00170 | c[a][l]ing | callllllng |
| 4 | 0.00196 | a[c]ep[t] | acceptttttttt |
| 5 | 0.00197 | fa[l][i]ng | fallllllng |
| 6 | 0.00217 | hi[l]ar[y] | hilllllaryy |
| 7 | 0.00227 | m[i][s][i]ng | missssssssssing |
| 8 | 0.00271 | ba[n]e[d] | banedddddddddd |
| 9 | 0.00277 | t[h][r][e] | threeeeeeeee |
| 10 | 0.00302 | th(er) | therrrreeeee |

TABLE A2. Bottom 10 (nonzero) kernels by an alternate normalized entropy, $H_{alt}$.

well balanced, all of the weight is on the well balanced short ones when randomly picking tokens. However, as people create longer stretched versions of (pa), they tend to use more 'a's than 'p's, and near perfect balance is not maintained. This is better captured by the measure $H = 0.80982$.

As our main measure of balance, we chose the view better representing how well balanced tokens are as they are stretched, equally weighing lengths. This does have the limitation that groups of tokens with different lengths have different sizes, and some of them may contain a single token, possibly increasing the variance of the measure. It is possible this could be improved in the future by only including lengths that have a certain number of examples, or possibly creating larger bins of lengths for the longer tokens like we do in the balance plots.

We include the same plots and tables for $H_{alt}$ as we did with $H$, and many of the observations are similar. Fig. A2 shows the two jellyfish plots for $H_{alt}$. Similar to before, Fig. A2A is the version containing all words and for Fig. A2B we remove the words that have a value of 0 for entropy. The top of the plots in Fig. A2 shows the frequency histograms in each case. As before, after removing kernels with an entropy of 0, we see a small left-shift in the highest ranked kernels, and then the distribution largely stabilizes. Again, the highest ranked kernels tend to be more equally balanced, and kernels only stretching a single character tend to be lower ranked.

Table A1 shows the kernels with the ten largest entropies and Table A2 shows those with the ten smallest nonzero entropies as measured in this alternate way. We observe that the kernels with largest entropies are all of the form $(l_1 l_2)$ and are almost perfectly balanced given the view of equally weighing all tokens. The kernels with lowest entropies all expand to regular words that when spelled in the standard way contain a letter that is repeated, plus these kernels allow other letters to stretch.

Finally, Fig. A3 shows the scatter plot of each kernel where the horizontal axis is given by this alternate measure of balance, $H_{alt}$, and the vertical coordinate is again given by the measure of stretch for the kernel using the Gini coefficient, $G$. We again see that the kernels span the two dimensional space.

We still get the same kind of rough vertical banding that we saw in Fig. 9 for the same reason, but we also see a curved dense band at lower entropy values, which seems to mostly contain kernels whose base word is spelled with a double letter, like 'summer' (with kernel [s][u][m][e][r]).

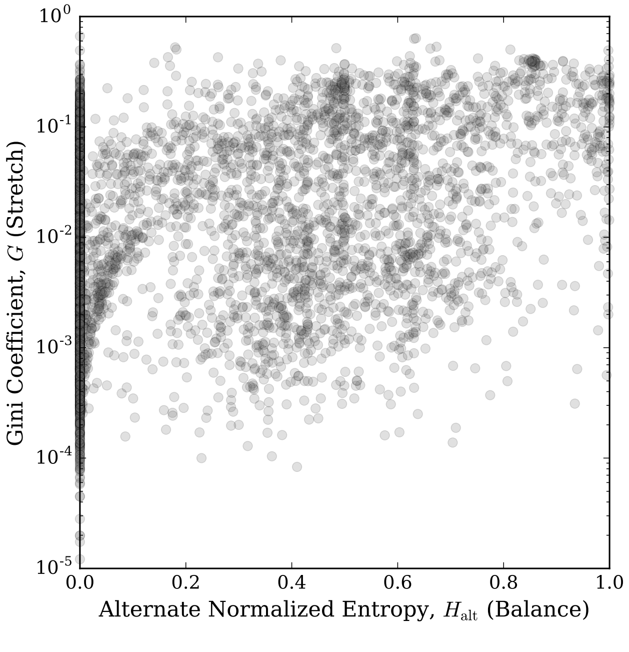


FIG. A3. Kernels plotted in Balance-Stretch parameter space using an alternate measure of normalized entropy for balance. Each kernel is plotted horizontally by the value of its balance parameter, given by an alternate normalized entropy, $H_{alt}$, and vertically (on a logarithmic scale) by its stretch parameter, given by the Gini coefficient, $G$, of its token count distribution. Larger entropy implies greater balance and larger Gini coefficient implies greater stretch.
