# Supplementary material for: Hahahahaha, Duuuuude, Yeeessss!: A two-parameter characterization of stretchable words and the dynamics of mistypings and misspellings
Source: PLoS One. 2020 May 27;15(5):e0232938. doi: 10.1371/journal.pone.0232938 (PMC7252599; doi:10.1371/journal.pone.0232938)
Supplement: S2 Appendix — (PDF) [file pone.0232938.s002.pdf]

Appendix B: Stretch Ratio

For each kernel, we also measure a 'stretch ratio', ρ. This is simply the ratio of the total number of stretched tokens, n_s, to the total number of unstretched tokens, n_u, for that kernel. That is,

$$\rho = \frac{n_s}{n_u}. \quad \text{(B1)}$$

Fig. B1 gives the jellyfish plot for the stretch ratio. Like Fig. 8, the horizontal axis has a logarithmic scale and the histogram bins have logarithmic widths. The stretch ratio distribution stays fairly stable across ranks, except for the highest ranked kernels, which tend to have a larger ratio.

This stretch ratio can be thought of as a simple measure for the stretchiness of a kernel, with a larger ratio representing stretchier words. As stretched versions of the word are used more, the numerator increases and the ratio value increases. Conversely, as unstretched versions of the kernel are used more, the denominator increases, and the ratio value decreases. However, this simpler measure uses less information from the full distribution than a measure like the Gini coefficient does, so we would expect some differences between the two. Indeed, Fig. B2 shows that there are some kernels for which the two measures seem to disagree. Yet, Fig. B2 shows that the stretch ratio and Gini coefficient are quite well correlated, with Pearson correlation coefficient 0.89 ($p < 10^{-100}$), so there is not much gained by including both. We choose to use the Gini coefficient as our main measure of stretchiness both because of its wide usage and because of the fact that it uses more information from the full distribution than the simpler stretch ratio.

Table B1 shows the top 10 kernels by stretch ratio and Table B2 gives the bottom 10. The correlation between stretch ratio and Gini coefficient, at least for the least stretchy kernels, can be seen further when comparing this

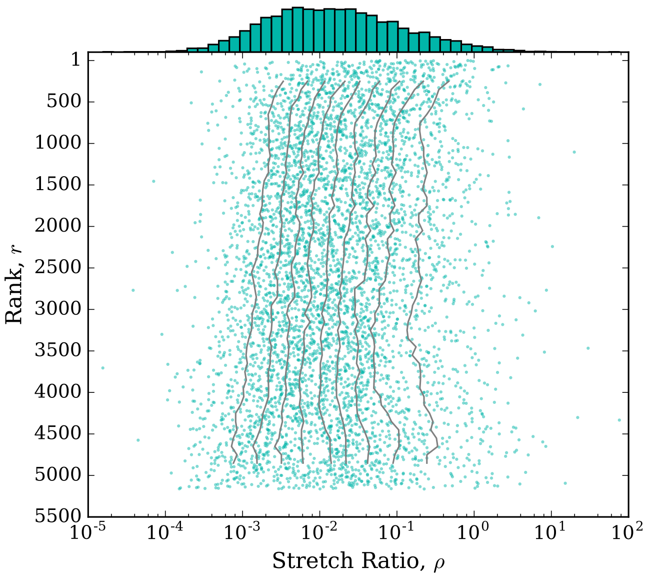

FIG. B1. Jellyfish plots for kernel stretch ratio, ρ, as given by the ratio of the sum of the kernel's stretched tokens to the sum or its unstretched tokens. The histogram is given at the top of the plot (with logarithmic width bins). Kernels are plotted vertically by their rank and horizontally (on a logarithmic scale) by their stretch ratio. The deciles $0.1, 0.2, \ldots, 0.9$ are calculated for rolling bins of 500 kernels and are plotted as the 'tentacles'.

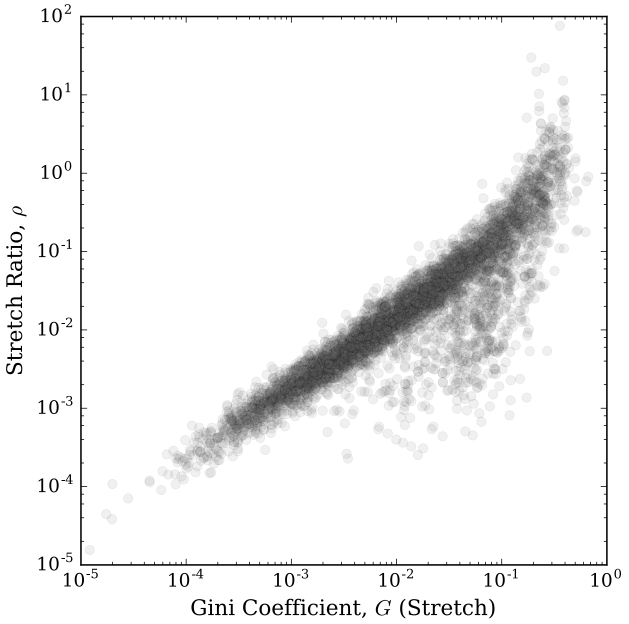

FIG. B2. Scatter plot of measures of stretch for each kernel. For each kernel, the horizontal axis gives its stretch as measured by the Gini coefficient, G, of its token count distribution and the vertical axis gives its stretch ratio, ρ. Both axes have a logarithmic scale.

	ρ	Kernel	Example token
1	76.04717	s[o][c][o][r][o][k]	socorrokkkkkk
2	29.94863	mou(ha)	mouhahahaha
3	21.93369	p[f](ha)	pffhahahaha
4	19.82821	bu(ha)	buhahahahaha
5	15.15702	(ha)j(ah)(ja)(ha)	hahahahajahajaha
6	10.32701	pu(ha)	puhahahahaa
7	8.63055	(ha)(ba)(ha)	habahahhaha
8	8.47429	(ha)b(ha)	hahahhahabha
9	8.13269	(ah)j(ah)	ahahahjahah
10	7.72953	a[e]h[o]	aehoooooooooooooo

TABLE B1. Top 10 kernels by stretch ratio, ρ.

	ρ	Kernel	Example token
1	0.00002	am[p]	ampppppppppp
2	0.00004	fr[o]m	frooooooooom
3	0.00004	m[a]kes	maaaaaaakes
4	0.00007	w[i]th	wiiiiiiiiiiiiiith
5	0.00009	eve[r]y	everrrrrrrrrrry
6	0.00011	p[r]a	prrrrrrrrrrrra
7	0.00011	watch[i]ng	watchiiiing
8	0.00011	s[i]nce	siiiiiiiince
9	0.00012	pla[y]ed	playyyyyyyed
10	0.00012	vi[a]	viaaaaaaaaaaaaaaa

TABLE B2. Bottom 10 kernels by stretch ratio, ρ.

to Table V. Many of the kernels that show up as the least stretchy words (lowest Gini coefficients) also show up here in the list of kernels with smallest stretch ratio.
