# Supplementary material for: Hahahahaha, Duuuuude, Yeeessss!: A two-parameter characterization of stretchable words and the dynamics of mistypings and misspellings
Source: PLoS One. 2020 May 27;15(5):e0232938. doi: 10.1371/journal.pone.0232938 (PMC7252599; doi:10.1371/journal.pone.0232938)
Supplement: S3 Appendix — (PDF) [file pone.0232938.s003.pdf]

Appendix C: "Drawing presentable trees" algorithmic bugs

Wetherel and Shannon presented an algorithm for drawing large trees in a nice way in their paper "Tidy drawing of trees" [38]. The article "Drawing presentable trees" [39] by Mill and related code [40], based largely on the earlier work of Wetherel and Shannon, provide a version of the algorithm written in the Python syntax, but both the article and the code contain algorithmic bugs. In the following, we present the bugs we found.

We will discuss Listing 5 in Mill's paper [39], as that is the version that most closely resembles Algorithm 3 of Wetherel's and Shannon's paper [38], which is what our code to create the spelling trees is based off of.

In Listing 5, the definition of `setup` contains the code:

```
elif len(tree.children) == 1:
    place = tree.children[0].x - 1
```

This needs to be split into a left case and a right case. If the only child node is a left child, then the parent should be placed to the right by one, and if the only child node is a right child, then the parent should be placed to the left by one. The `DrawTree` class needs a way to tell if a node has a left or right child. Let us assume the class `DrawTree` has an attribute `left` properly implemented that is set to `True` iff the node has a left child. Then the code should be something more like the following:

```
elif len(tree.children) == 1:
    if tree.left:
        place = tree.children[0].x + 1
    else:
        place = tree.children[0].x - 1
```

Compare the above fix to the corresponding code in the `right_visit` case in the first `while` loop in Algorithm 3 in "Tidy drawing of trees" [38]:

```
elseif current↑.left_son = nil
    then place := current↑.right_son↑.x - 1;
elseif current↑.right_son = nil
    then place := current↑.left_son↑.x + 1;
```

Later in Listing 5 in the definition of `setup` is the following line:

```
nexts[depth] += 2
```

However, we want the next available spot, recorded in `nexts`, to be two spots to the right of the current placement, and the current placement is sometimes different from the current next available spot. Thus the line should look something like the following:

```
nexts[depth] = tree.x + 2
```

Again, compare this to the corresponding code found near the end of the `right_visit` case of the first `while` loop of Algorithm 3 in "Tidy drawing of trees":

```
next_pos[h] := current↑.x + 2;
```

The final bug in Listing 5 is not an algorithmic bug, but merely a typo. In the definition of `addmods` is the line of code:

```
modsum += tree.offset
```

However, `tree` does not have the attribute `offset`. Instead the `mod` attribute should be added to the accumulated sum as follows:

```
modsum += tree.mod
```
